# Integrated tendon repair and staged skin grafting for a severe forearm degloving injury: A case report from a low-resource setting

**DOI:** 10.1097/MD.0000000000051328

**Published:** 2026-10-23

**Authors:** Garuun Mohamed Abdikadir, Moise Mvuye Manishimwe, Mahmoud Bakheet Abdulrahman, Fadlalla Ali Khalid, Chinyere Nkemjika Anyanwu, Emeka Godson Anyanwu

**Affiliations:** a Department of Orthopedic Surgery, Kampala International University Teaching Hospital, Ishaka Bushenyi, Uganda; b Department of Microbiology and Immunology, Faculty of Biomedical Sciences, Kampala International University, Ishaka Bushenyi, Uganda; c Department of Anatomy, Faculty of Biomedical Sciences, Kampala International University, Ishaka Bushenyi, Uganda.

**Keywords:** forearm degloving, low-resource setting, skin graft, tendon repair, trauma reconstruction

## Abstract

**Rationale:**

Upper-limb degloving injuries are uncommon but can cause substantial functional impairment, particularly when accompanied by tendon injury. Their management is especially challenging in resource-constrained settings where microsurgical reconstructive options may be limited.

**Patient concerns:**

A 21-year-old, right-handed man presented 2 hours after a road traffic collision with an extensive degloving injury involving the right forearm, wrist, and dorsal thumb.

**Diagnoses:**

Examination and operative exploration demonstrated exposed underlying structures and rupture of the flexor digitorum superficialis tendon slips. Radiographs also revealed a concomitant distal humerus fracture.

**Interventions:**

Following resuscitation, the patient underwent urgent wound exploration, extensive irrigation and debridement, primary tendon repair, and partial wound closure. The residual dorsal thumb defect was subsequently covered with a split-thickness skin graft harvested from the right thigh. The distal humerus fracture was managed conservatively with immobilization.

**Outcomes:**

The postoperative course was uncomplicated. At 6 weeks, the wounds and skin graft had healed satisfactorily, thumb and finger movements were preserved, and the patient had regained useful hand function following physiotherapy. The concomitant distal humerus fracture remained under conservative management with immobilization during this period.

**Lessons:**

Early debridement and tendon repair combined with staged split-thickness skin grafting can provide satisfactory wound coverage and early functional recovery in selected upper-limb degloving injuries when microsurgical reconstruction is unavailable.

## 1. Introduction

Degloving injuries are high-energy soft-tissue injuries in which the skin and subcutaneous tissue are separated from the underlying fascia, muscle, tendon, or bone. They most commonly result from road traffic collisions or industrial accidents that generate tangential shearing forces.^[1-3]^ Disruption of the perforating blood vessels may render the avulsed tissue nonviable and increase the risks of necrosis, infection, and functional impairment.^[1,3]^ Although degloving injuries constitute a relatively small proportion of trauma admissions, their consequences can be substantial, particularly when the upper limb is involved.^[3,4]^

Forearm degloving injuries present particular reconstructive challenges because of the close anatomical relationship among the tendons, nerves, vessels, and bones. Management requires early assessment of tissue viability, thorough irrigation and debridement, repair of injured functional structures, and appropriate soft-tissue coverage. These injuries may be accompanied by fractures or tendon injuries; 1 study from Sudan reported associated fractures or tendon injuries in more than 60% of upper-limb degloving cases.^[2]^ Available reconstructive options include primary closure, use of the degloved skin as a graft, split-thickness skin grafting, pedicled flaps, and free-tissue transfer, depending on the wound characteristics, exposed structures, tissue viability, and available expertise.^[2,5]^ Treatment decisions may be especially challenging in resource-constrained settings where microsurgical reconstruction and negative-pressure wound therapy are not consistently available.

This report describes the staged management and early functional outcome of a severe forearm degloving injury involving rupture of the flexor digitorum superficialis (FDS) tendon slips and a concomitant distal humerus fracture in a young adult. Management comprised urgent debridement, primary tendon repair, partial wound closure, conservative fracture treatment, and delayed split-thickness skin grafting. The case illustrates a context-adapted approach to restoring wound coverage and hand function when advanced reconstructive options are limited.

## 2. Case history and presentation

### 2.1. Patient demographics and mechanism of injury

A 21-year-old, right-hand dominant male bicyclist presented to the emergency department about 2 hours after a high-speed road traffic accident. He was cycling when he was struck in the back by a truck, resulting in a high-energy degloving injury of the right upper limb. The effect and subsequent debridement on the asphalt caused an avulsion of skin and subcutaneous tissue on the anterolateral side of the right forearm which extended into the wrist and over the dorsal part of the thumb with underlying structures exposed (Fig. 1). The patient was a university student who did not receive any serious first aid at the scene other than bystanders putting pressure to contain the bleeding. He had no known chronic medical condition, previous surgery involving the affected limb, or regular medication use. Primary and secondary trauma surveys did not identify any additional injuries.

**Figure 1.**
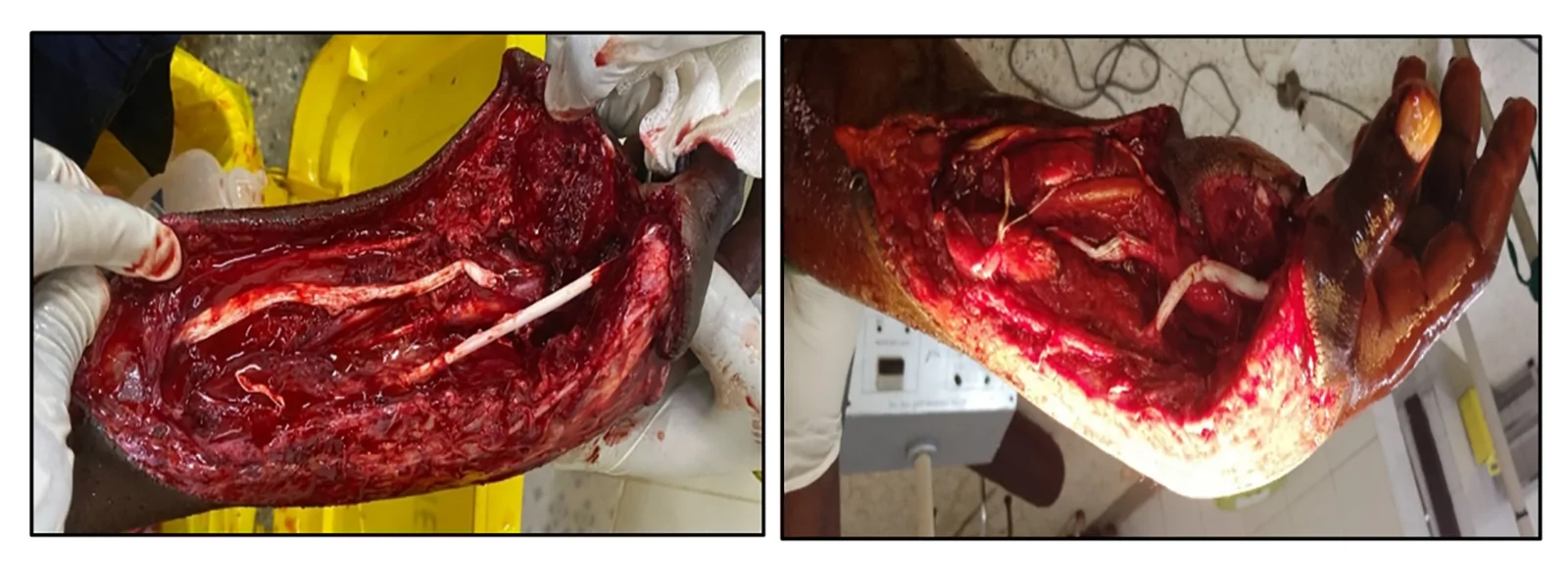
Appearance of the injury site at admission.

### 2.2. Injury circumstances

The collision generated tangential shearing forces that avulsed the skin and subcutaneous tissues of the right forearm. The patient fell onto his right side, and the forearm scraped against the road surface, resulting in a large contaminated degloving wound with irregular edges and exposed underlying tendons. The injury was classified clinically as an open, non-circumferential degloving injury. Because of its irregular configuration, the total wound surface area was not formally measured at presentation; however, after partial primary closure, the residual dorsal thumb defect measured approximately 4 × 3 cm.

### 2.3. Initial presentation and examination

At presentation, the patient was alert and hemodynamically stable, with a heart rate of 98 beats/min and blood pressure of 125/80 mm Hg, but was in severe pain. Primary and secondary trauma surveys revealed no clinically apparent injury other than to the right upper limb. Examination of the distal arm revealed deformity, swelling, and crepitus suggestive of a distal humerus fracture (Fig. 2A). A large, open, non-circumferential degloving wound involved the volar-radial aspect of the distal forearm and extended across the wrist to the dorsum of the hand at the base of the thumb. The wound exposed the underlying forearm musculature and flexor tendons, with visible disruption of the FDS tendon slips. The dorsal soft tissues over the proximal phalanx of the thumb were also deficient. Despite the extensive soft-tissue injury, distal perfusion was preserved: capillary refill was brisk, and both radial and ulnar pulses were palpable. Gross sensation was intact in the median, ulnar, and radial nerve distributions. The patient could move his fingers weakly, although thumb movement was limited by pain and soft-tissue loss. Active wound bleeding during transport was controlled with direct-pressure dressings.

**Figure 2.**
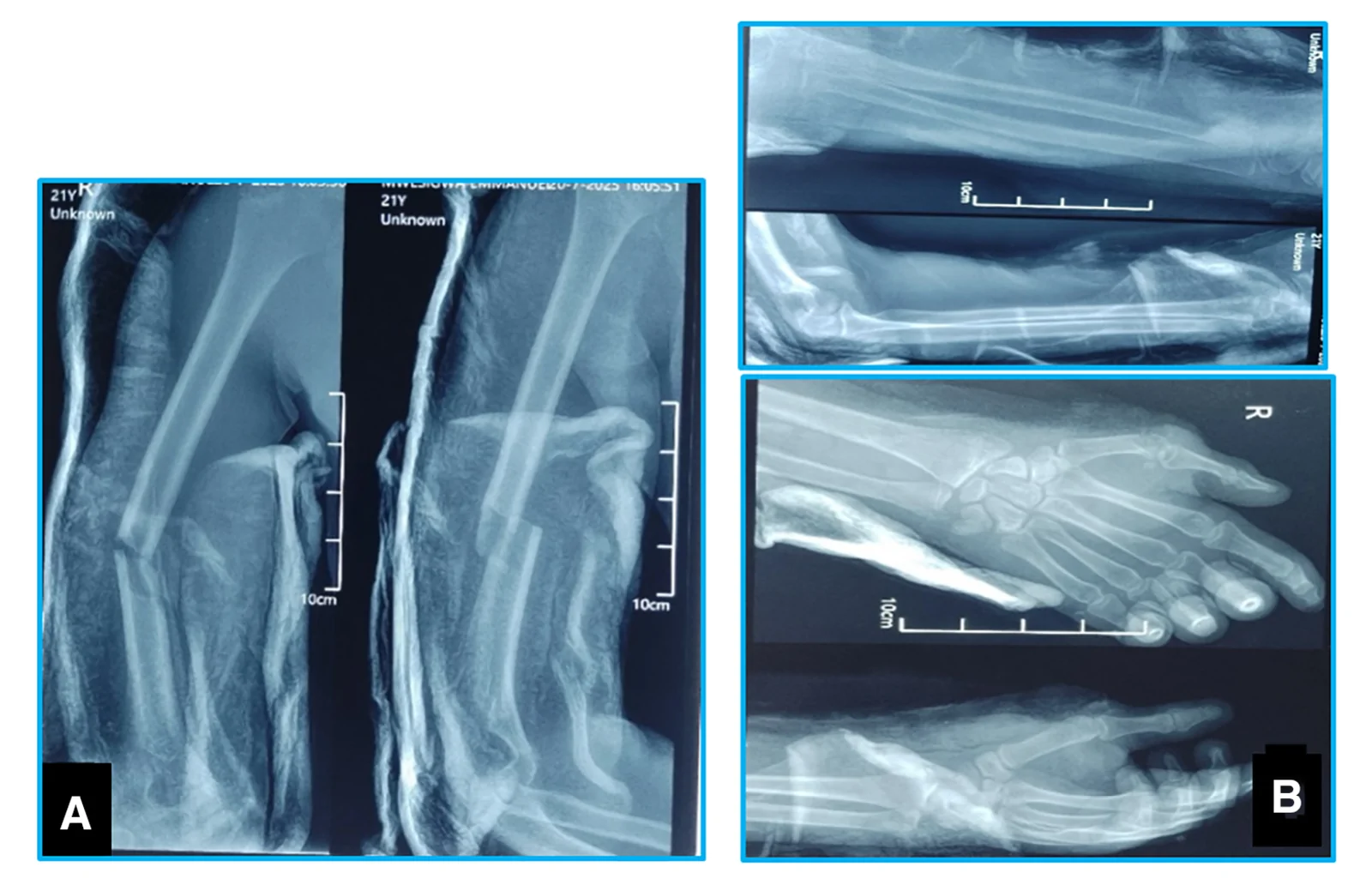
(A) Figure shows x-rays of the right arm, while (B) figure shows those of the right forearm and the right hand.

### 2.4. Imaging and investigations

Radiographs demonstrated a comminuted fracture of the distal humerus (Fig. 2A). The fracture was closed, with no communication between the fracture site and the distal forearm degloving wound. The x-rays of the forearm and hand (Fig. 2B) revealed no apparent fracture of any bones of the radius, ulna, or hand, and no radiopaque foreign material in the wound. The shoulders and elbow joints were normal except for the humeral fracture. Trauma blood tests were normal (hemoglobin 13 g/dL, white blood cell count 9 × 10^9^/L), and the patient’s tetanus vaccination was current. With intact peripheral pulses, further vascular imaging, such as computed tomography (CT) angiography, was not considered necessary. There was no sign of compartment syndrome or other limb-threatening disorder besides the degloving itself.

### 2.5. Initial emergency management

Within the emergency room, pain management and wound care were given priority. Short-term analgesia with intravenous morphine and prophylactic administration of a broad-spectrum antibiotic (first-generation cephalosporin) was initiated because of the high level of contamination risk. Tetanus prophylaxis was used (tetanus toxoid injection intramuscularly). Direct pressure and limb elevation were used to control the hemorrhage. The degloved injury was liberally sprayed with normal saline to clear road debris and then put under sterile saline-impregnated dressings. The fracture in the distal humerus was supported using a posterior U-slab (plaster splint) that was applied between the upper arm and the forearm to keep the elbow in flexion and the wrist in a neutral position. The affected upper limb was elevated above heart level to minimize swelling. During resuscitation and wound preparation, care was taken to prevent additional injury to exposed tendons and neurovascular structures.

### 2.6. Operative management

The patient was brought to the operating room on an emergency basis, approximately 4 hours post-injury, to have a complete wound exploration, debridement, and repair. The wound was extended under general anesthesia and tourniquet control to allow access and extensively debrided – all obviously devitalized skin edges and nonviable subcutaneous tissue were removed. The wound bed was once more irrigated using large amounts of normal saline. Intraoperative observation revealed a full rupture of the FDS tendon of the distal forearm (with index and middle finger slips) and partial tear of the surrounding muscle fibers. The ruptured FDS tendon slips to the index and middle fingers were repaired end-to-end using a modified Kessler core-suture technique with 2-0 polypropylene, reinforced with a running 6-0 nylon epitendinous suture. The rest of the tendons (such as flexor digitorum profundus and flexor pollicis longus of the thumb) were intact but were closely inspected and irrigated. No significant nerve transections were identified, and both radial and ulnar arteries were patent on inspection/palpation. Once tendon repair had been completed, the focus shifted to soft-tissue closure. Since the surface area of degloved skin was large, only a partial primary closure could be done: the proximal and distal wound edges on the forearm were moved and brought as near as possible to cover the musculature and were sewn with interrupted nylon sutures. This covered a majority of the volar forearm structures. Nevertheless, some part of the wound on the back of the thumb (around 4 × 3 cm on the proximal phalanx) was unable to be stitched together, mainly because of the loss of skin (Fig. 3A). This region was thus left as an open granulating wound; it was then covered with non-adherent moist dressings to provide skin coverage later. The distal humerus fracture was managed conservatively because it was closed and maintained satisfactory alignment in the splint. Following surgery, the arm was re-immobilized in a U-slab while leaving the hand and fingers free to permit movement and facilitate early rehabilitation.

**Figure 3.**
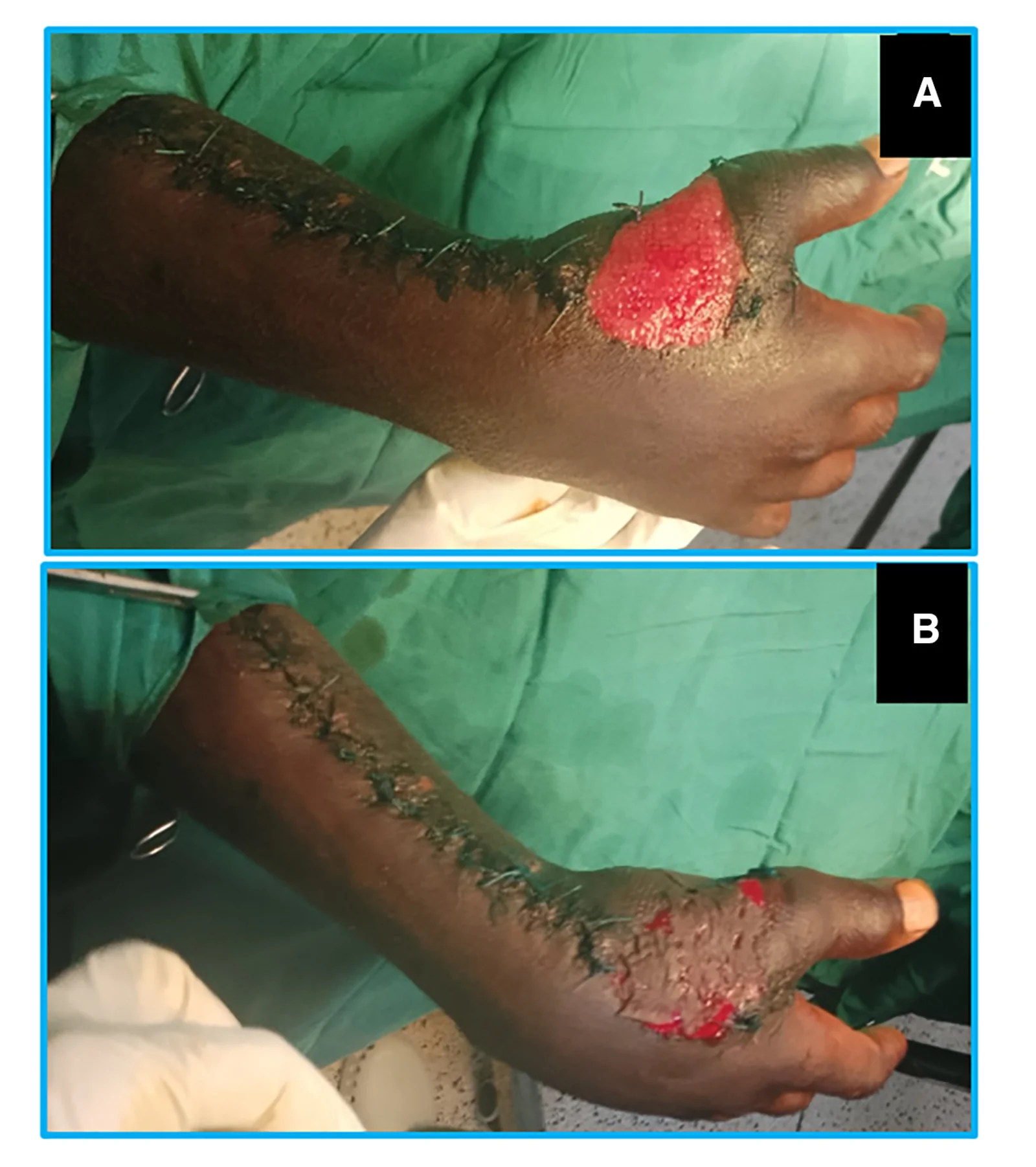
Showing the aspect of the wound after 3 weeks of soft tissue repair: (A) before skin graft and (B) after skin graft.

*Postoperative course*: Following the initial operation, the patient was transferred to the surgical ward in stable condition. Five days of antibiotic treatment using intravenous cefazolin was continued to treat the skin flora (due to the contamination), and analgesics (alternating paracetamol and ibuprofen, and using opiates when necessary) were administered to treat pain. The right hand was retained at the level above the heart. The forearm wound did not show any signs of infection or compartment syndrome in the first 48 hours. The repaired FDS tendon was assessed cautiously during the early postoperative period. The patient demonstrated gentle active finger flexion and extension, suggesting preservation of the repair and overall flexor function. The flexor digitorum profundus tendons had been confirmed to be intact during operative exploration. Early rehabilitation was initiated under the physiotherapy team’s supervision, with guidelines for the first 48 to 72 hours including the beginning of passive range-of-motion exercises of the fingers, which prevent stiffness and adhesion of the repaired tendon. The patient was taught to flex and extend the interphalangeal joints of the fingers with caution within the limits of pain, and the wrist and elbow were immobilized. One week later, under splint protection, active finger movement under gentle control was promoted.

During the 3 weeks, the wound was treated by frequent dressing changes. The open cavity on the dorsum of the thumb was covered with a saline gauze, and thereafter with an antiseptic ointment, to induce the development of sound granulation tissue. By week 3 after injury, the granulation bed was clean and well-vascularized (Fig. 3A), which was appropriate to definitively cover the skin. This patient was then brought back to the theater where he received a split-thickness skin graft (STSG). A split-thickness skin graft that was dermatome-harvested was taken from the right thigh at a ratio of 1.5:1. The defect on the dorsal thumb was covered with this graft. Following grafting, the limb was immobilized in a U-slab, and the graft was secured with sutures and a tie-over dressing (Fig. 3B). The forearm and wrist areas of the wound were already being epithelialized at the edges or were healing by secondary intention beneath the dressings. The patient remained hospitalized throughout wound preparation and skin grafting, with a total inpatient stay of approximately 4 weeks.

### 2.7. Outcome and follow-up

Primary inspection of the graft dressing occurred 7 days after grafting: the STSG was almost complete, with new healthy skin observed on the thumb (Fig. 4). The patient showed intact finger and thumb movement – he could flex/extend the right thumb at the interphalangeal and metacarpophalangeal joints, and could make a loose fist with the fingers. The early mobilization protocol was helping to glide the repaired flexor tendon well. The fingers were slightly stiff, but with continuous hand therapy (the physiotherapist instructed the patient in tendon-gliding exercises and active flexion-increasing exercises), the symptoms improved. The wounds of the forearm and hand were completely healed at around 6 weeks following the injury (3 weeks following grafting, Fig. 5). The thumb graft was a durable and sensory skin graft. Surgical sutures of the graft harvest site and thumb were taken off. The distal humerus fracture was managed with immobilization in a U-slab for 6 weeks. Further orthopedic follow-up was planned to assess fracture healing clinically and guide the gradual introduction of elbow mobilization and rehabilitation. The patient demonstrated useful hand function at the 6-week follow-up. He could oppose the thumb to the fingers, grasp small objects, and actively extend all digits. There was no infection or wound breakdown. Formal grip-strength testing was deferred because the distal humerus fracture remained under orthopedic management. The fracture was managed with immobilization in a U-slab for 6 weeks. Further orthopedic follow-up was planned to assess fracture healing clinically and guide the gradual introduction of elbow mobilization and rehabilitation. At this stage, the patient was independent in feeding and grooming activities using the affected hand.

**Figure 4.**
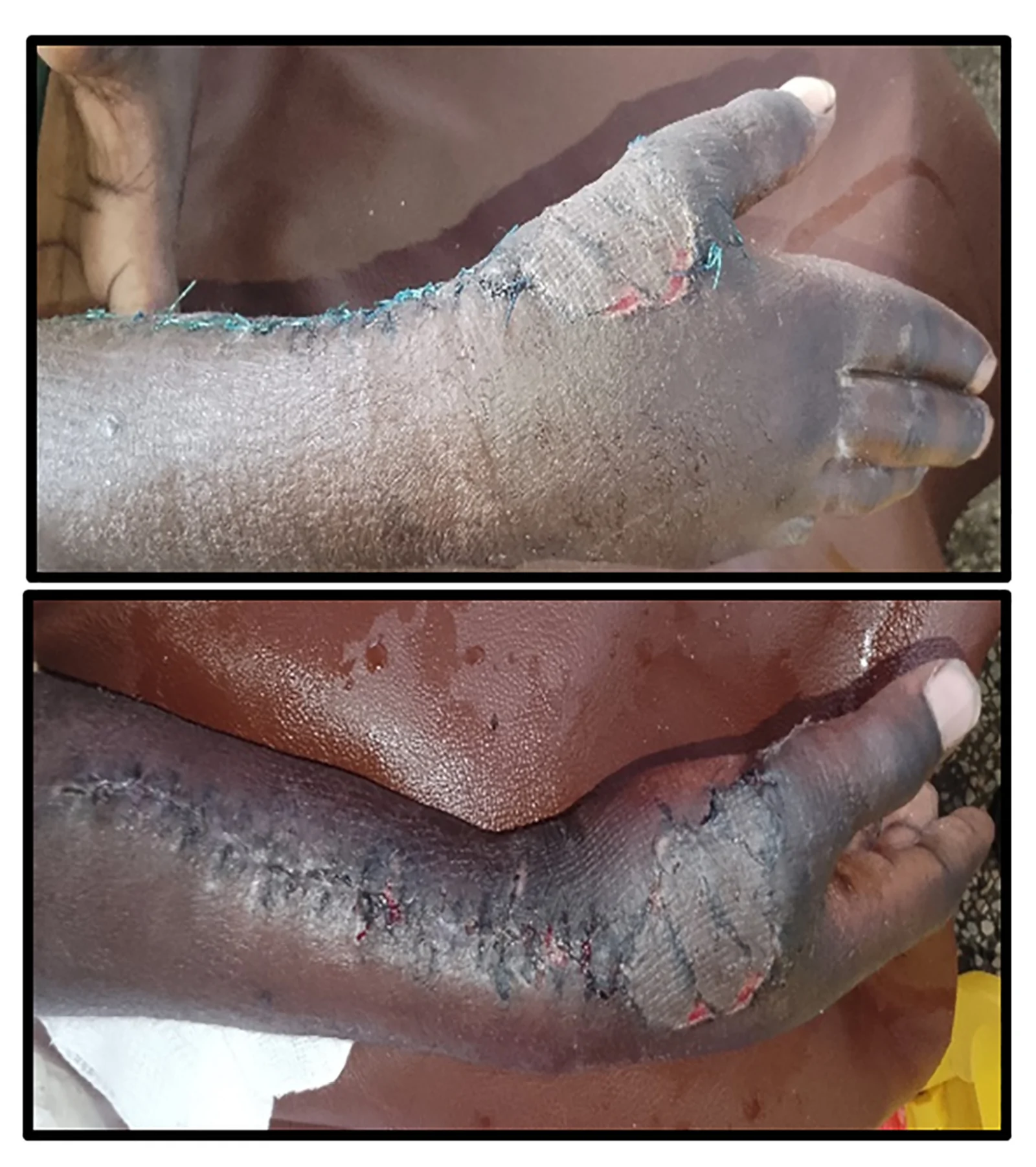
Aspect of the wound after 4 weeks of soft tissue repair, 1 week after skin graft.

**Figure 5.**
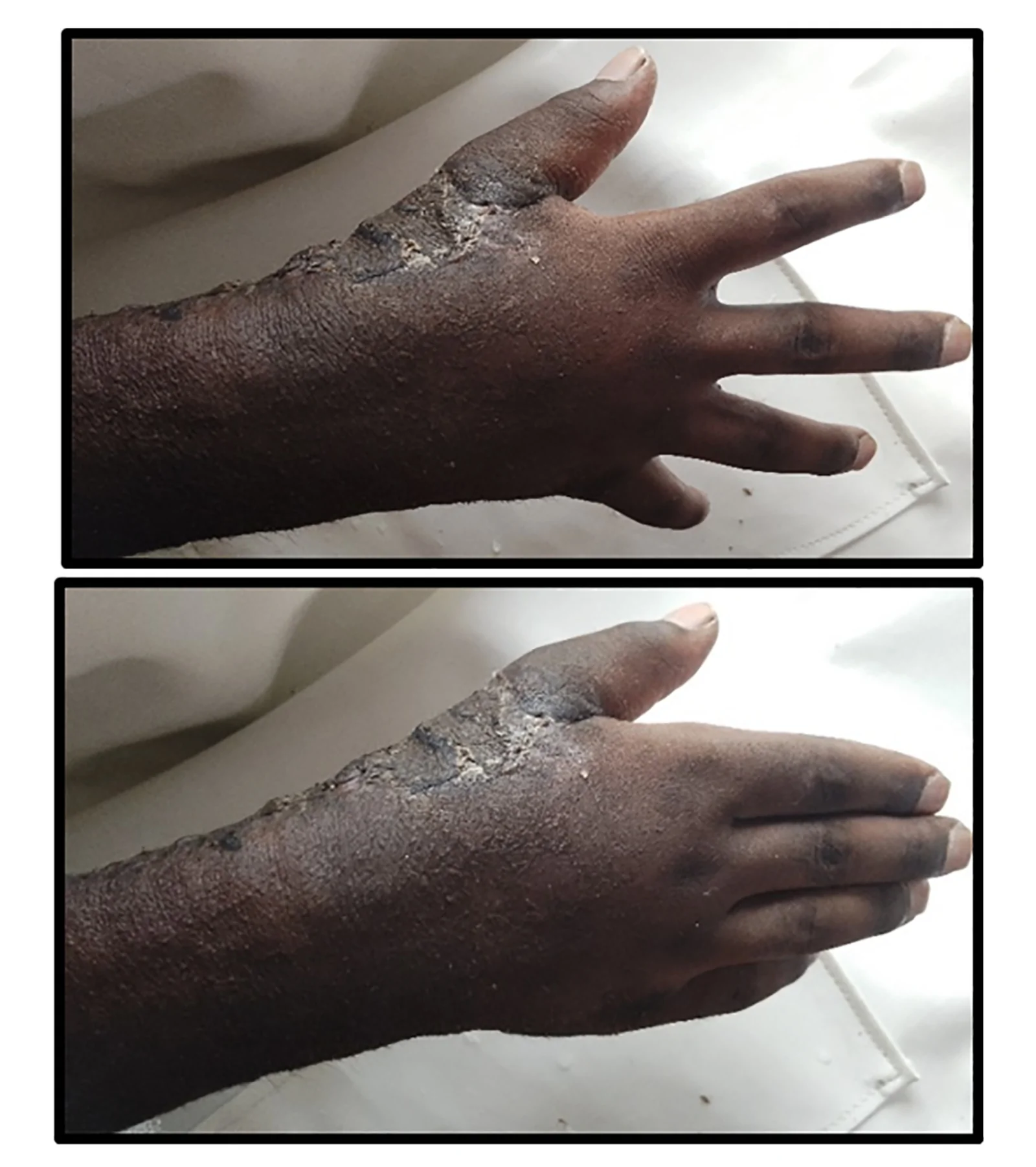
Aspect after 6 weeks of soft tissue repair, 3 weeks after skin graft.

## 3. Discussion

The case illustrates the challenges of dealing with a severe forearm degloving injury with tendon involvement and fracture in a young patient in a resource-limited environment. The main problems were infection control, structural maintenance, and the attainment of a permanent wound cover to restore limb function. This case concerns a healthy 21-year-old cyclist who was hit by a truck and received high-energy trauma that is characteristic of upper-limb degloving mechanisms.^[6]^ These injuries occur mostly in low- and middle-income countries among young males, but they represent more widespread trauma epidemiology in Africa and Asia.^[2,7]^ Severe shearing of the skin and subcutaneous tissue occurs, separating it and the fascia, tearing perforating vessels and leaving devascularized foci vulnerable to necrosis and infection.^[6]^ Here, the exposure of the tendons and the fact that there was a fracture of the distal humerus added to the complexity as well as the rehabilitation requirements of the surgery.

### 3.1. Pathophysiology and injury pattern

Degloving injuries are high-energy avulsion injuries and, in many cases, involve deep structural injury. Such injuries are especially devastating in the forearm, where the soft-tissue envelope and a high concentration of tendons, nerves, and vessels are located. In the volar forearm, the flexor tendons and the neurovascular bundles are located, whereas the extensor tendons and the skin are thinner on the dorsal surface. After degloving, the skin flaps lose their viability because of the absence of perforating blood flow,^[6]^ According to Ali and Mohamed,^[2]^ 61 percent of upper-limb degloving injuries in Sudan were associated with fractures, which was also observed in our case. These combined injuries are associated with a long stay in the hospital and an increased risk of infection.^[8,9]^ The awareness of this connection makes it clear that early, multidisciplinary management that incorporates orthopedic and reconstructive skills is necessary.

### 3.2. Urgent surgical management

The timeliness of interventions is essential to positive results in degloving injuries. Early irrigation and debridement eliminate contaminants, restrict bacterial growth, and define viable tissue. Wound exploration and tendon repair were done in this patient within hours of injury. At the index procedure, primary tendon repair maintained motion and avoided tendon retraction – a methodology in line with the principles of hand surgery and endorsed by Hudson,^[10]^ and Voche.^[11]^ They both highlight the importance of early tendon repair in clean wounds to enhance functional results and avoid adhesion formation. Orthopedic and soft-tissue coordinated management was also necessary. The distal humeral fracture was conservatively treated to reduce the risk of infection by hardware in an otherwise potentially contaminated area. This is a staged method in which soft-tissue salvage is considered before definitive bone work is done, and is very much in line with the idea of trauma care in the case of contaminated limb injury.

### 3.3. Wound coverage strategy in a resource-limited environment

Soft-tissue coverage is a central component of degloving-injury management. In well-resourced settings, extensive upper-extremity defects with exposed vital structures may require microsurgical free-tissue transfer using flaps such as the anterolateral thigh or latissimus dorsi flap.^[11-13]^ Pedicled local or regional flaps may also provide vascularized coverage when tendons, nerves, vessels, or bone remain exposed. However, these procedures require specialized expertise, equipment, and perioperative support that may not be readily available in resource-constrained hospitals. Reapplication of viable avulsed skin after defatting as a full-thickness skin graft (FTSG) is another potential option and has produced satisfactory coverage in selected cases.^[6]^ Although this option was considered, the avulsed skin in the present case was heavily contaminated, extensively abraded, and judged nonviable during debridement; it was therefore unsuitable for grafting and was discarded. Negative-pressure wound therapy (NPWT) may facilitate exudate control, wound-bed preparation, and granulation before definitive coverage; however, it was not used because the necessary equipment and consumables were unavailable at the treating facility.

A pragmatic 2-stage strategy was therefore adopted. Primary closure was performed where tissue approximation was possible, while the residual dorsal thumb defect was managed with serial dressings. Because the wound was initially contaminated and required further assessment of tissue viability, definitive coverage was delayed until a clean, well-vascularized granulation bed had developed. At 3 weeks, the approximately 4 × 3 cm residual defect was considered suitable for split-thickness skin grafting. A pedicled or free flap was not considered necessary at this stage because the relatively small defect had developed an adequate vascularized wound bed for grafting. This approach achieved stable coverage while avoiding the additional donor-site morbidity and technical demands associated with flap reconstruction. Yildirim et al^[14]^ similarly reported satisfactory functional recovery following split-thickness skin grafting for forearm degloving injuries. In the present case, graft take was nearly complete at the first inspection 1 week after grafting, indicating satisfactory wound-bed preparation and infection control.

### 3.4. Postoperative care, rehabilitation, and outcome

Postoperative management is essential in maximizing results. Infection is likely avoided by elevation of the limbs and broad-spectrum antibiotics, which occurs in up to 25 percent of open degloving wounds.^[9]^ The first postoperative week was devoted to physiotherapy, which started with passive exercises and proceeded to active exercises after 2 weeks, which is in line with the evidence that early movement facilitates tendon gliding and decreases stiffness.^[15]^ The patient had good thumb and finger mobility with slight stiffness by the sixth week, which indicated that the tendons were still functional, and the healing process was satisfactory. The fact that he was able to resume his normal daily functions was an indication of early functional restoration, which is a good sign of eventual complete recovery. These findings are similar to those of Hudson et al^[10]^ who stressed that compliance in the upper-limb rehabilitation process is an essential factor in improving the final outcomes.

### 3.5. Comparison with literature and lessons learned

We consider our management to be in line with the international evidence that supports early multidisciplinary intervention, careful debridement, and context-appropriate reconstruction. The advantages of a staged approach with early debridement, tendon stabilization, and subsequent soft-tissue coverage, as noted by Voche et al,^[11]^ are the main factors that can be identified in this case. Similarly, Lekuya et al^[9]^ suggested that reconstructive teams should be involved early in degloving injuries that are fractured. In our case, a lack of microsurgical capacity forced the use of traditional methods of reconstruction, but the results were similar to those of other more resource-rich environments. The success highlights the fact that doing the basics, such as timely debridement, meticulous tendon repair, and well-planned grafting, will result in functional and aesthetic success even in the absence of advanced facilities. Also, this case supports the rule of replacing like with like and balancing the possibilities and resources.^[2]^

### 3.6. Limitations and reflections

Our patient at 6 weeks had stable wound coverage and almost full hand mobility. The graft provided good contour and durability to the dorsal thumb, and no infection or contracture was reported. These findings are in line with reports that young patients are able to restore good functioning after limb-salvage procedures, provided they are addressed in a timely manner.^[2]^ The principal limitations of this report include the short follow-up period, the absence of follow-up radiographs to objectively assess fracture union, and the lack of standardized functional assessment using measures such as grip-strength testing or the Disabilities of the Arm, Shoulder and Hand score. Long-term follow-up will be required to assess possible complications such as scar contraction or tendon adhesion. However, the current result indicates that anatomically informed intervention, implemented early and with well-planned rehabilitation, can lead to satisfactory results even in an environment limited by resources.

## 4. Conclusion

The case solidifies the main principles of degloving injury treatment, including early debridement, timely tendon repair, and proper soft-tissue coverage. Even where there is a lack of microsurgical capacity, good limb salvage and function are possible by following sound reconstructive principles and staged skin grafting. The patient’s recovery demonstrates the possibility of obtaining positive results through strict surgical discipline, interdisciplinary organization, and timely physiotherapy. The wider use of such practical strategies would enhance the results of such injuries in low-resource areas.

## Author contributions

**Conceptualization:** Garuun Mohamed Abdikadir, Moise Mvuye Manishimwe, Mahmoud Bakheet Abdulrahman, Fadlalla Ali Khalid, Chinyere Nkemjika Anyanwu, Emeka Godson Anyanwu.

**Data curation:** Garuun Mohamed Abdikadir, Moise Mvuye Manishimwe, Fadlalla Ali Khalid, Chinyere Nkemjika Anyanwu.

**Formal analysis:** Mahmoud Bakheet Abdulrahman, Chinyere Nkemjika Anyanwu, Emeka Godson Anyanwu.

**Investigation:** Garuun Mohamed Abdikadir, Moise Mvuye Manishimwe, Mahmoud Bakheet Abdulrahman.

**Project administration:** Emeka Godson Anyanwu.

**Supervision:** Fadlalla Ali Khalid.

**Visualization:** Garuun Mohamed Abdikadir, Moise Mvuye Manishimwe, Fadlalla Ali Khalid, Emeka Godson Anyanwu.

**Writing – original draft:** Garuun Mohamed Abdikadir, Moise Mvuye Manishimwe, Mahmoud Bakheet Abdulrahman, Chinyere Nkemjika Anyanwu, Emeka Godson Anyanwu.

**Writing – review & editing:** Fadlalla Ali Khalid, Chinyere Nkemjika Anyanwu, Emeka Godson Anyanwu.
